# Low Concentrations of the Solvent Dimethyl Sulphoxide Alter Intrinsic Excitability Properties of Cortical and Hippocampal Pyramidal Cells

**DOI:** 10.1371/journal.pone.0092557

**Published:** 2014-03-19

**Authors:** Francesco Tamagnini, Sarah Scullion, Jonathan T. Brown, Andrew D. Randall

**Affiliations:** 1 School of Physiology and Pharmacology, University of Bristol, Bristol, United Kingdom; 2 Institute of Biomedical and Clinical Sciences, Medical School, University of Exeter, Exeter, United Kingdom; University of California, Berkeley, United States of America

## Abstract

Dimethylsulfoxide (DMSO) is a widely used solvent in biology. It has many applications perhaps the most common of which is in aiding the preparation of drug solutions from hydrophobic chemical entities. Recent studies have suggested that this molecule may be able to induce apoptosis in neural tissues urging caution regarding its introduction into humans, for example as part of stem cell transplants. Here we have used *in vitro* electrophysiological methods applied to murine brain slices to examine whether a few hours treatment with 0.05% DMSO (a concentration regarded by many as innocuous) alters intrinsic excitability properties of neurones. We investigated pyramidal neurones in two distinct brain regions, namely area CA1 of the hippocampus and layer 2 of perirhinal cortex. In the former there was no effect on resting potential but input resistance was decreased by DMSO pre-treatment. In line with this action potential count for any level of depolarizing current stimulus was reduced by ∼25% following DMSO treatment. I_h_-mediated “sag” was also increased in CA1 pyramids and action potential waveform analysis demonstrated that DMSO treatment moved action potential threshold towards resting potential. In perirhinal cortex a decreased action potential output for various depolarizing current stimuli was also seen. In these cells action potential threshold was unaltered by DMSO but a significant increase in action potential width was apparent. These data indicate that pre-treatment with this widely employed solvent can elicit multifaceted neurophysiological changes in mammalian neurones at concentrations below those frequently encountered in the published literature.

## Introduction

There is frequently a requirement to use non-aqueous solvents in biological experiments, for example, to dissolve pharmacological agents that have a limited aqueous solubility. This manipulation is usually performed by making a concentrated stock solution in 100% solvent that is subsequently diluted into aqueous media to generate a final solution for application to the cells, tissue or organism under investigation. Good experimental design dictates that the drug-treated group is then compared to a group treated with only the vehicle containing solution. What is less frequently considered, however, is what effects do the vehicle containing solutions produce in their own right.

In biology, the organosulphur, polar, aprotic molecule dimethylsulphoxide (DMSO) has become unquestionably the most widely employed solvent, at least for *in vitro* studies. For example, the individual chemical constituents of large compound collections used for high throughput screening in the pharmaceutical industry are universally prepared in DMSO, unless there is some specific reason not so to do [1]. Although, perhaps without strong evidential reasons, it has seemingly become a general rule of thumb in biological folklore that concentrations of 0.1% (v/v) DMSO or lower are generally biologically innocuous, whereas concentrations above 1% are likely to be highly undesirable. As well as being used as solvent, another major use of DMSO in biology is in the cryopreservation of tissues- a use that leads to the introduction of considerable amounts of DMSO into humans in clinical scenarios. DMSO has also been used to enhance cell fusion events and also to manipulate cell permeability. Many of these actions are mediated through the interaction of DMSO with the lipid constituents of biological membranes [2].

Here we have used *in vitro* brain slice neurophysiology methods to examine if and how a period of DMSO treatment alters the core intrinsic excitability (IE) properties of mammalian neurones. We performed our analyses of two classes of neurone. The first was the hippocampal CA1 pyramidal cell (CA1-PC), probably the mammalian brain's most commonly studied neuronal type [3]. In addition, we investigated cortical pyramidal neurones in layer 2 of the perirhinal cortex (PR-L2PC), a cell type proposed to play a pivotal role in recognition memory [4]. We studied the effects of 0.05% DMSO (v/v) a concentration of ∼7 mM, which is half that employed in very many biological studies. Contrary to widespread opinion we find that this solvent concentration is not experimentally inert, but generates significant changes to the IE of pyramidal cells in both brain regions, effects which persist beyond the period of exposure.

## Methods

### Experimental animals

Male C57BL/6J mice aged 4-5 weeks were used for all experiments. These animals were group housed and maintained on a standard 12∶12 hour light/dark cycle with access to food and water *ad libitum*.

### Preparation of brain slices

Preparation of hippocampal slices was performed as previously described [5]. All animal procedures were approved by the local ethical committee of the University of Bristol and were in accordance with schedule 1 of the UK Animals (Scientific Procedures) Act (1986). In brief, mice were sacrificed by cervical dislocation and the brain was rapidly removed and transferred to an ice cold (∼4°C), sucrose-based slicing solution comprising (in mM): sucrose, 189; D-glucose, 10; NaHCO_3_, 26; KCl, 3; MgSO_4_, 5; CaCl_2_, 0.1; NaH_2_PO_4_, 1.25, continuously bubbled with carbogen (95% O_2_, 5% CO_2_). The cerebellum and frontal and dorsal parts were removed with a single scalpel cut. The sample was then mounted on a metal plate ventral side up and 300 μm thickness horizontal sections were prepared using a Leica VT1200 vibratome.

Perirhinal slices of 300 μm thickness were also prepared in a sucrose-based slicing medium and using the same Leica vibratome employed for hippocampal sections. These slices were isolated from “modified coronal” sections cut at an angle 45 degrees to the dorsal-ventral axis.

After sectioning, slices were submerged in a storage vessel which contained our standard artificial cerebrospinal fluid (aCSF) consisting of (in mM): NaCl, 124; KCl, 3; NaHCO_3_, 26; CaCl_2_, 2; NaH_2_PO_4_, 1.25; MgSO_4_, 1; D-glucose, 10 and equilibrated with 95% O_2_ and 5% CO_2_. The slices were gradually heated to ∼32–34°C for 30 minutes, after which they were stored at room temperature for one hour prior to being treated with either DMSO (0.05% v/v) or no added compound for 2–5 hours prior to recording; the slices obtained from each animal were exclusively allocated to either one or the other of the two described treatments. For the hippocampus, for example, 19 slices from 13 animals were used for the control group and 13 slices from 8 animals for the DMSO treated group. For neurophysiological analysis an individual slice was transferred to a submersion style recording chamber mounted on an Olympus BX51 fixed stage microscope. The chamber was continuously perfused (∼2 ml.min^−1^) with standard gassed aCSF. The temperature of the slice was maintained at 33±1°C by an in-line solution heating device coupled to a feedback control circuit. The extracellular solution used during recording did not contain DMSO, irrespective of the prior treatment, thus any actions of DMSO we observed were not an acute effect arising from the presence of DMSO but a consequence of the previous period of prior exposure.

### Electrophysiological methods

IE properties were studied using single cell patch clamp recording from either CA1-PC or PR-L2PC. The recording methods employed are very similar to those we used for our previous studies of intrinsic properties of CA1-pyramidal cells in Aβ-overproducing transgenic mice and healthy aged animals [5] [6]. Neurones were visually identified using infra-red differential interference contrast optics. Pipettes were fabricated from borosilicate glass and were fire polished such that their resistance was 2.5–4.5 MΩ when filled with pipette solution. For perirhinal recordings the pipette solution consisted of (mM): K-gluconate, 145; NaCl, 5; K-HEPES, 10; EGTA, 0.2; Na-GTP, 0.3; Mg-ATP, 4; pH 7.3, 285–290 mOsm. For hippocampal recordings the electrode solution was a slightly modified version consisting (mM) K-gluconate, 135; NaCl, 5; K-HEPES, 10; EGTA, 0.2; Na-GTP, 0.3; Mg-ATP, 4; biocytin 13.4; Alexafluor 488, 0.02; pH 7.3, 285–290 mOsm.

After forming gigaseals and entering the whole cell configuration in voltage-clamp mode, the amplifier was immediately switched to bridge-mode current-clamp in which all experiments were performed. The pairing of our pipette solution and aCSF produces a liquid junction potential error of 15 mV; this was corrected for arithmetically in all data sets. All recordings were made using a MultiClamp 700B amplifier (Molecular Devices, Union City, CA). Data were lowpass filtered (5–10 kHz) and then digitised (20–100 kHz) and stored on a personal computer using pClamp10 electrophysiology software.

### *In vitro* electrophysiology protocols and data analysis

Analysis of current-clamp recordings, including action potential waveform analysis was carried out with custom-written routines within the Matlab environment. Resting potential (V_rest_) was measured as soon after starting recording as possible. Following determination of resting potential for all other measurements the unstimulated membrane potential was set to −80 mV using the appropriate amount of current injection. This manipulation was applied because certain intrinsic properties, for example input resistance and sag, are voltage-dependent.

Membrane resistance was analysed in multiple ways. The first measure (R_in-exp_) assessed resting input resistance independently of the “sag”-producing I_h_ activation that occurs during hyperpolarizing current steps. This was calculated using Ohm's law from the amplitude of an infinite time extrapolation of a single exponential curve fitted to the membrane charging response generated by a −100 pA current injection. The exponential fit was made between points at 10 and 95% of peak negative amplitude. This exponential fit was also used to determine membrane time constant and to determine one measure of the extent of sag (see below). The second measure of input resistance (R_in-ss_), includes the contribution from additional I_h_ activation produced during the current step. This was calculated using Ohm's law by determining the steady-state (post-sag) voltage deflection produced by a 500 ms −100 pA stimulus. In CA1-PC, the input resistance at -80 mV (R_in-slope_) was also measured (as the reciprocal of slope conductance) using linear regression of the steady state voltage responses elicited by a series of 8 low amplitude (−50 to+30 pA), 500 ms duration current steps. Hyperpolarization-activated sag was measured in two ways. The first measurement (sag_sub_) simply expressed the difference between the peak and steady state hyperpolarizations produced by a 500 ms −100 pA current injection as a percentage of the peak hyperpolarization. The second measure of sag (sag_fit_) measured the decay in response relative to the amplitude of the infinite time extrapolation used to determine R_in-exp_. In addition to sag, the amplitude of the sag-related rebound depolarization was also measured relative to the pre-stimulus membrane potential. Measurements of impedance were made as previously described [7]. Briefly, oscillating subthreshold voltage responses were evoked by a sinusoidal current injection of constant amplitude (between ±20 pA and ±50 pA) of linearly increasing frequency starting at 0.5 Hz and rising to 20 Hz over a period of 30 s. The impedance (sZ) *versus* frequency profile was derived by dividing the fast Fourier transform of the voltage response by that of the applied sinusoidal current stimulus: Z (MΩ) = V(fft)/I(fft). The quality factor of the oscillator was calculated as the ratio between the impedance at the peak frequency and the impedance at 1 Hz: (Q = Z_peak_/Z_1 Hz_).

To study suprathreshold behaviours such as firing rates and patterns and action potential waveforms, depolarizing current injections of 500 ms duration were used to elicit action potential (AP) firing. In CA1-PC these varied in amplitude stepwise from +50 to +300 pA in 50 pA increments, whereas in PR-L2PC, which have a lower input resistance, a larger stimulus range (50–600 pA) was employed to ensure action potential generation was seen in all cells. From these data sets the relationship between the stimulus amplitude and the number and pattern of APs elicited was examined. Initially to assess individual AP waveforms the first spike fired by a 300 pA (CA1-PC) or 600 pA (PR-L2PC) current injection was analysed. In addition, for hippocampal recordings we also analysed the properties of the first AP in the first sweep in which one or more AP was observed (which was usually a stimulus below 300 pA). AP threshold was determined from phase plots as the voltage at which dV/dt surpassed 15 V.s^−1^ [8], [9]. Spike width was measured at −15 mV which is approximately halfway between threshold (∼−60 mV) and action potential peak (∼+30 mV).

Each parameter investigated was compared between the two experimental groups using a two tailed unpaired Students t-test or two way ANOVA, as appropriate.

## Results

### Effects of DMSO incubation on CA1 pyramidal cells

**Figure 1:**
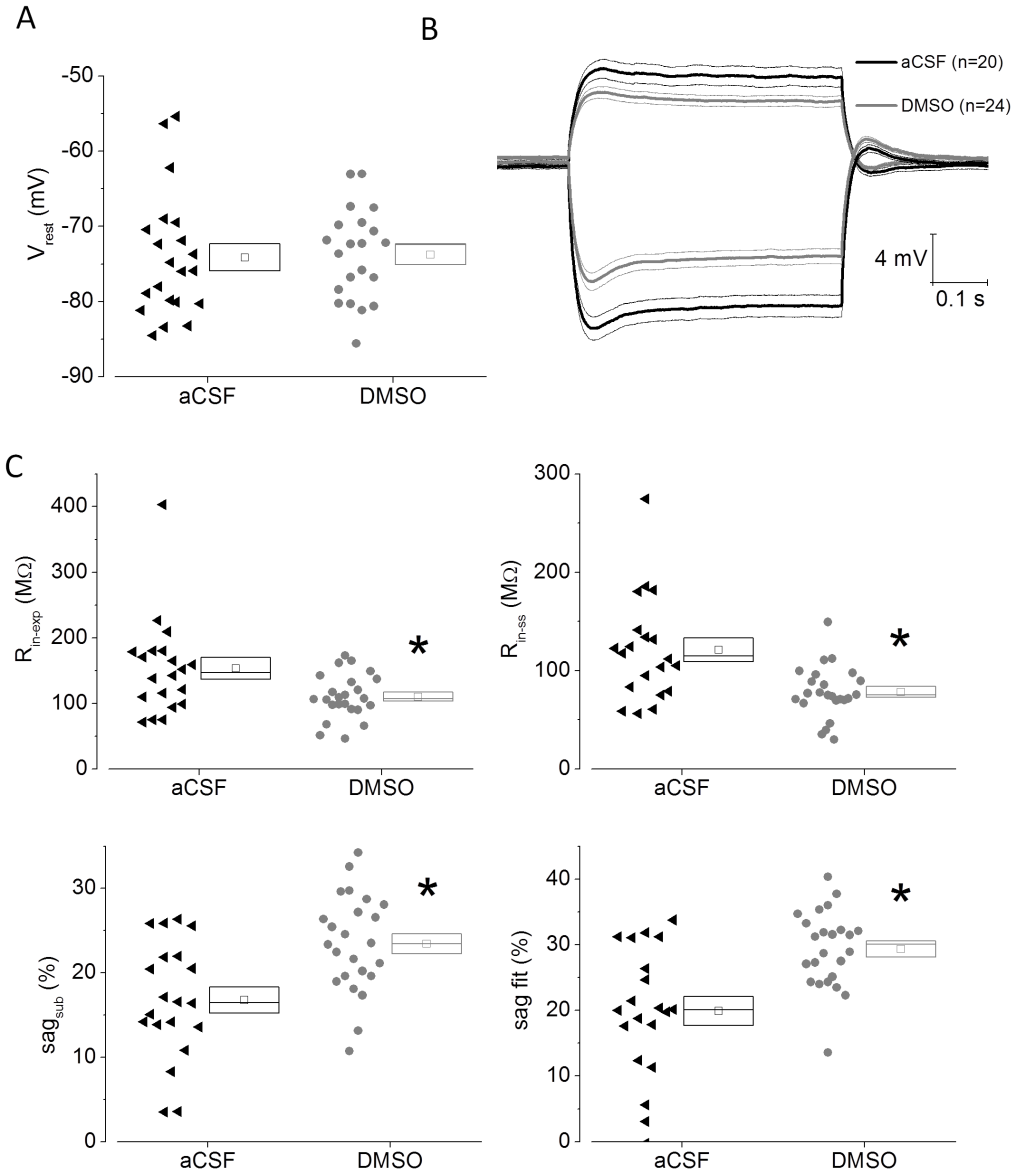
DMSO pre-treatment modifies subthreshold intrinsic properties in CA1-PC. A) A scatter/box plot of zero current potential recorded from control and DMSO pre-treated CA1-PC. In this and all other similar plots, the symbols to the left represent data from individual neurones, whereas the box to the right plots the mean (central symbol) plus the upper and lower bounds of the standard error and the median. In this and all other figures data from control neurones are presented in black and data from DMSO pre-treated cells are shown in grey. B) A plot of the average voltage response to both -100 (downwards) and +50 pA (upwards) current stimuli applied to CA1-PC. The thicker central line corresponds to the mean whereas the two adjacent thinner lines represent the bounds encompassed by 1 standard error of the mean. C) Scatter plots of sub-threshold intrinsic properties derived from −100 pA stimuli applied at a fixed membrane potential of −80 mV. Two measurements of input resistance and sag are presented (see methods).

The zero current potential observed soon after entering the whole cell mode (an indicator of resting potential) was not different when control and DMSO pre-treated CA1-PC were compared (Figure 1A). Although resting potentials were not different in the two groups, all further measurements were carried out at a set membrane potential of −80 mV to reduce variability arising from the range of resting potentials exhibited by each population (see Figure 1A).

When subthreshold intrinsic properties were measured using either positive or negative current injections significant differences between the control and DMSO pre-treated CA1-PC were clearly apparent. This is illustrated in Figure 1B which presents mean voltage responses to injections +50 pA and −100 pA compiled from 20 control and 24 DMSO treated cells.

Cell by cell analyses of intrinsic parameters derived from −100 pA current challenges are presented in Figure 1C. These confirm that there was a reduction of input resistance of over 30% in DMSO pre-treated cells. The solvent pre-treated cells also exhibited an enhanced fractional contribution of sag. This is perhaps unexpected given the smaller negative voltage deflection that occurred in these neurones for any current stimulus, which would typically reduce I_h_ channel activation. These differences in both input resistance (P<0.002) and sag were highly significant (P<0.0003), and remained significant when the single recording with the highest input resistance in the control group (which might be considered an outlier) was discounted.

Fitting an exponential decay function to the voltage trace between 10% and 95% of the total voltage drop upon the injection of a −100 pA current, allowed us to evaluate the membrane time constant τ on a cell by cell basis. In a RC circuit, such as the plasma membrane, τ = Rm.Cm, hence, from this analysis we have been able to obtain an estimation of Cm, by dividing τ by the extrapolated measure of membrane resistance described above (see methods).

**Figure 2:**
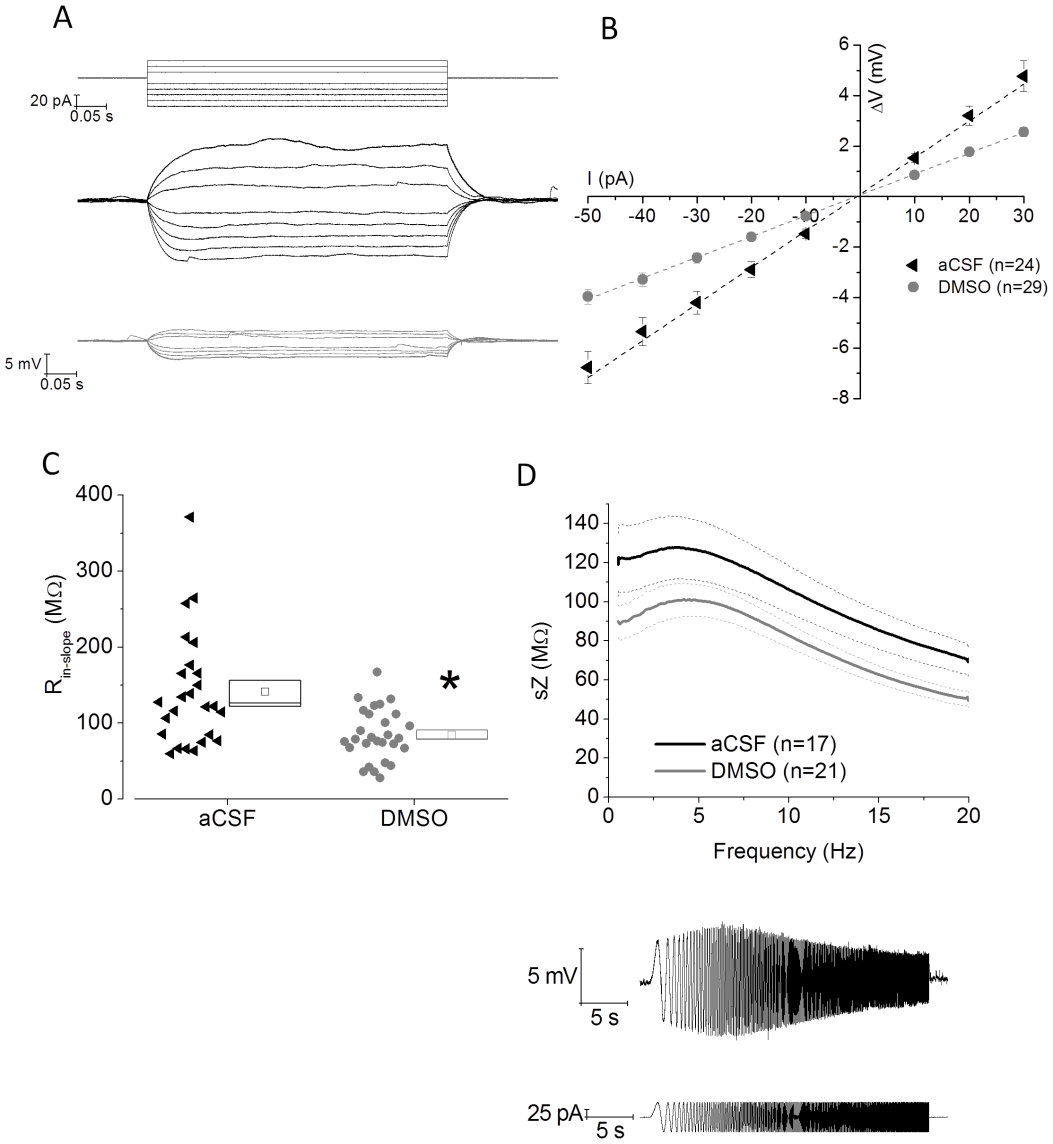
DMSO pre-treatment alters membrane resistance and impedance in CA1-PC. A) Voltage responses from an example control (middle, black) and DMSO pre-treated (bottom, grey) CA1-PC elicited by a series of 500 ms current stimuli varying in amplitude between −50 and +30 pA (top). B) Pooled data from a number of recordings like and including those shown in (A). The graph plots steady-state voltage deflection versus current stimulus. C) A scatter plot of input resistance derived from recordings like that in (A). Each symbol represents the slope-derived input resistance derived from a straight line fit through all the data points obtained from a single recording, the resistance in DMSO-treated cells was significantly lower (P<0.001) than in control cells. D) The top panel shows a plot of mean impedance *versus* stimulus frequency for control (black) and DMSO-treated (grey) CA1-PC. The thicker central line represents the mean values, and the dashed lines the bounds of 1 SEM. The bottom panel shows an example trace of the Vm of CA1 pyramidal cell resonating in response of the injection of a sinusoidal current injection of increasing frequency. The impedance Z(Ω) is measured as Z = V(fft)/I(fft). The quality factor of the resonator, Q, is calculated as the ratio between the Z at peak frequency and Z the frequency of 1 Hz (Q = Z_peak_/Z_1 Hz_).

As well as measurements based on analysis of responses to a single −100 pA hyperpolarizing current step, we also measured input resistance using an escalating series of low amplitude current injections, varying stepwise between −50 to +30 pA (Figure 2A). The pooled V-I relationships compiled from these are shown in Figure 2B. For each recording included in Figure 2B the value of the slope-derived input resistance (R_in-slope_) is presented in Figure 2C. This reiterates the significant change (P<0.001) in input resistance produced in CA1-PC by DMSO pre-treatment. The difference in Rm does not correspond to a difference in τ but in a significant difference in Cm, which is significantly higher in DMSO-treated slices compared to controls (Table 1).

**Table 1:** Comparison of passive membrane properties of control CA1 pyramidal neurons with those pre-treated with DMSO (0.05%).

	DMSO 0.05% n = 24		aCSF n = 20		P
	Mean	SEM	Mean	SEM	
**RMP (mV)**	−73.8	1.3	−73.8	1.9	0.99
**Rin-ss (MΩ)**	78.4	5.4	121.3	11.9	**0.001**
**Rin-exp (MΩ)**	110.3	6.9	153.3	16.5	**0.014**
**Rin-slope (MΩ)**	84.6	6.4	141.2	15.0	**0.0007**
**sag_sub (%)**	23.5	1.2	16.8	1.5	**0.001**
**sag_fit (%)**	29.4	1.2	19.9	2.2	**0.0003**
**tau (ms)**	14.3	0.9	13.1	1.1	0.4
**Cm fit (pF)**	140.9	11.6	99.6	12.9	**0.02**

Using current stimuli consisting of a sine wave linearly increasing in frequency (see Methods) we obtained measurements of impedance (i.e. resistance in the AC domain) versus frequency and therefore of the resonance properties of the cells in the two different conditions. This measure describes the property of the cell to either work as a low-pass pass filter (low Q, peak frequency close to 1 Hz) or as a high-pass filter (high Q, peak frequency >1 Hz). The resonance properties are mainly related to 2 voltage gated currents: a) I_M_, which is an hyperpolarising current activated upon depolarisation by the opening of voltage-gated potassium channels (VGKC) and that is selectively abolished by K_v7_ blockers, such as XE991; b) I_h_, which is a depolarising current activated upon hyperpolarisation by the opening of hyperpolarisation-cyclic nucleotide activated channels (HCN) and that is selectively blocked by HCN inhibitors such as ZD7288. The reciprocal role of I_M_ or I_h_ is strongly related to the resting membrane potential; in our conditions (Vm fixed at −80 mV) the I_h_ component prevails [7]. In the present study we observed a significant increase in the quality factor of the resonator (p = 0.01) after pre-treatment in DMSO, a non significant tendency towards increase in the maximal impedance, and no significant differences in the maximal frequency (Table 2). This result is consistent with the increase in sag observed in DMSO treated slices, which may underlie a role for DMSO in increasing I_h_.

**Table 2:** Comparison of intrinsic resonance properties of control CA1 pyramidal neurons with those pre-treated with DMSO (0.05%).

	DMSO 0.05% n = 24		aCSF n = 20		P
	Mean	SEM	Mean	SEM	
**Peak frequency (Hz)**	5.2	0.4	4.2	0.5	0.14
**Q**	1.2	0.03	1.1	0.02	**0.02**
**Peak Z**	105.8	9.3	131.6	16.6	0.16

**Figure 3:**
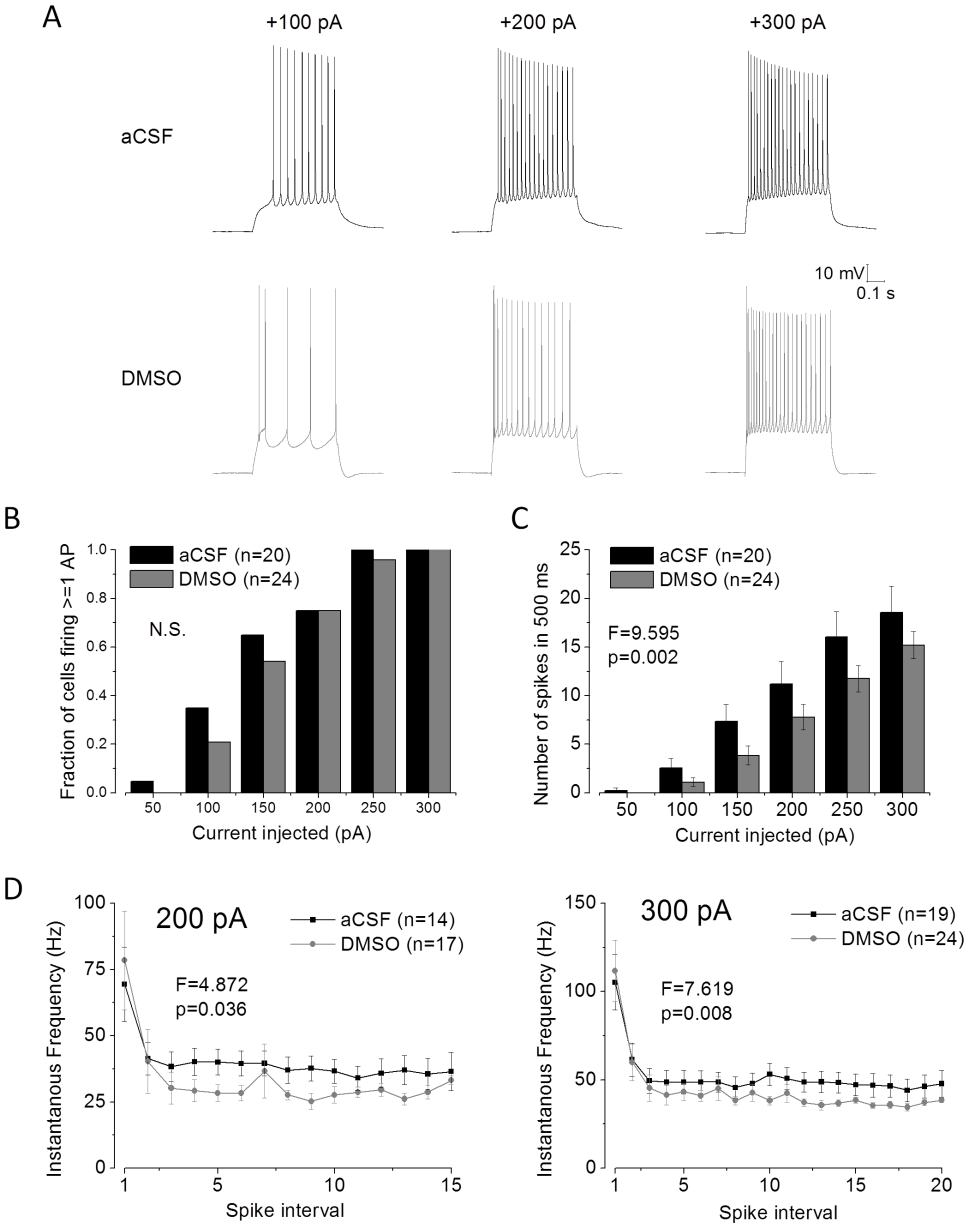
DMSO pre-treatment reduces action potential output in CA1-PC. A) Examples of the action potential firing produced by application of 500 ms depolarizing stimuli of 100 (left), 200 (middle) and 300 pA (right) amplitude applied at a pre-stimulus membrane potential of −80 mV. Data are show for both a control aCSF only CA1-PC (top) and a DMSO pre-treated CA1-PC (bottom). B) A plot of the percentage of recordings in which 1 or more action potential was elicited by the various amplitudes of current stimulus indicated on the ordinate. C) A plot of mean number of spikes versus amplitude of current stimulus for control and DMSO pre-treated CA1-PC. D) A plot of instantaneous action potential frequency versus spike interval for 500 ms current stimuli of 200 pA (left) and 300 pA (right) amplitude.

We also investigated action potential generation of CA1-PC cells stimulated with depolarizing current steps lasting 500 ms and varying in amplitude between 50 and 300 pA. Example traces from both groups are shown in Figure 3A. The fraction of cells firing one or more spikes for a given depolarizing stimulus is shown in Figure 3B. There is a suggestion here, at least with weaker stimuli, that it is less easy to produce firing in the DMSO pre-treated cells, as might be expected from their ∼30% lower input resistance. Reduced excitability in DMSO treated CA1-PC is also indicated by Figure 3C, which plots the number of spikes fired for any given stimulus; and demonstrates a clear reduction in total spikes fired for any stimulus after DMSO pre-treatment (P = 0.002, two way ANOVA). This reduction in spike number was also reflected in the temporal dynamics of spike firing. Thus, when instantaneous firing frequency was plotted for each successive spike interval, the first two, highest frequency, intervals were quite similar but the DMSO treated cells subsequently accommodated to a firing frequency around 20% lower than that observed in control cells. This is illustrated for the action potential firing dynamics in response to 200 and 300 pA stimuli in Figure 3D.

**Figure 4:**
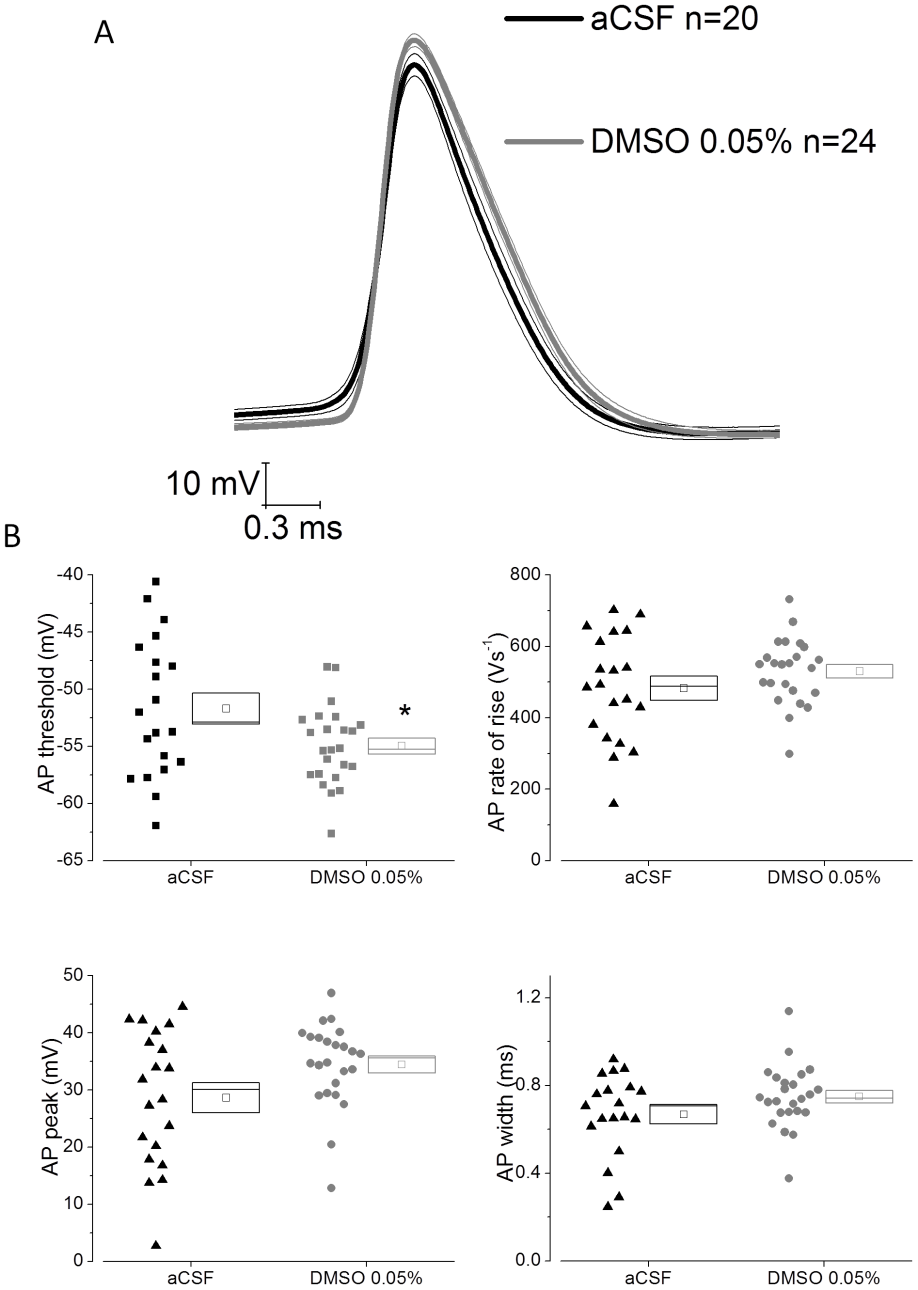
DMSO pre-treatment changes action potential threshold in CA1-PC. A) Averaged action potential waveform ± SEM from 20 control cells (aCSF) and 24 DMSO treated cells. The action potentials were the first spike to fire in response to a 300 pA depolarizing stimulus. B) Cell by cell analysis of action potential parameters for the action potentials used to compile (A). DMSO caused a negative shift in action potential threshold (P<0.03).

**Figure 5:**
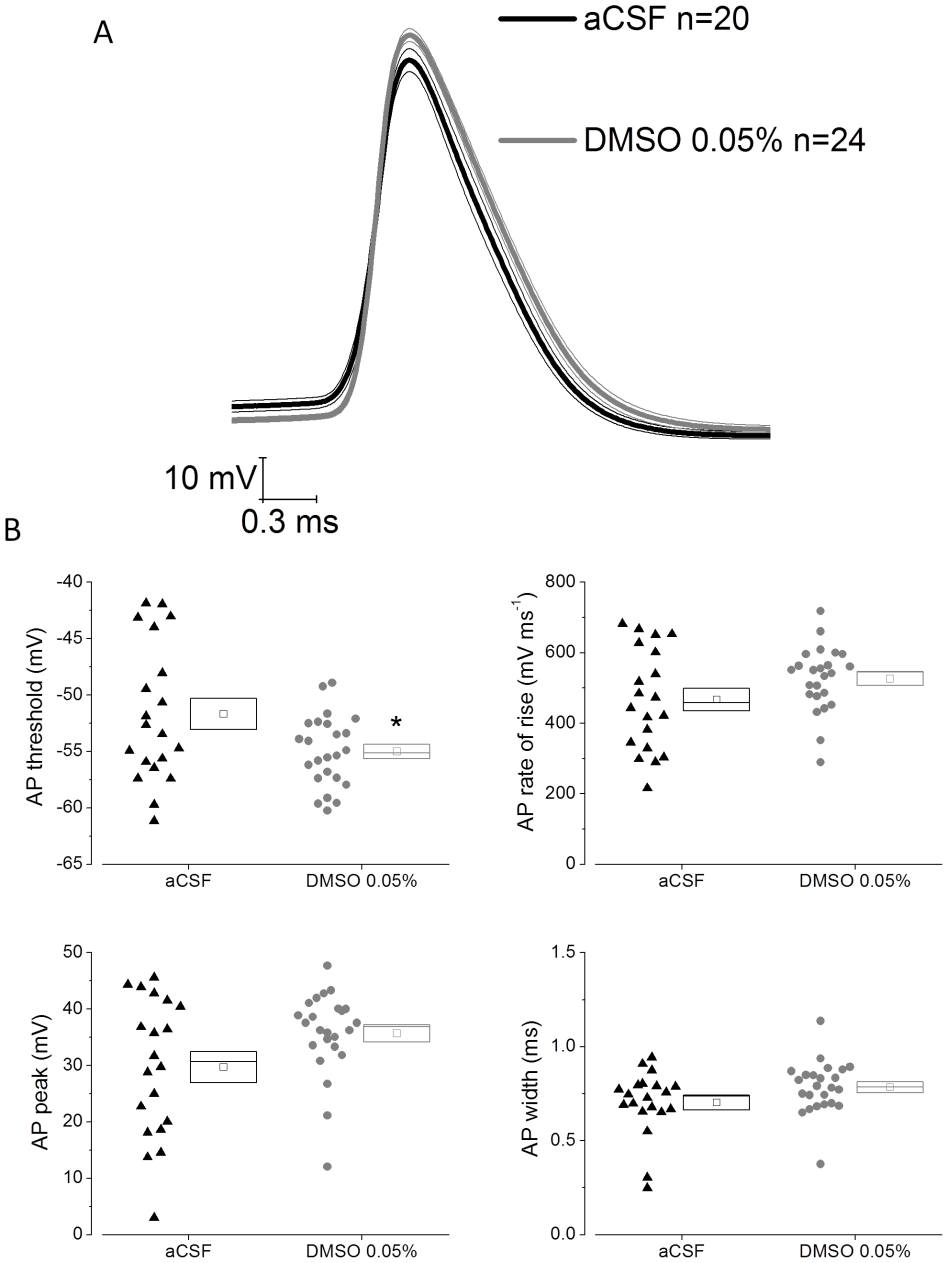
An alternative analysis of the action potential properties reveal the same effects of DMSO pre-treatment on the action potential threshold in CA1-PC. A) Averaged action potential waveform ± SEM from 20 control cells (aCSF) and 24 DMSO treated cells. The action potentials were the first spike to fire in response to the minimal depolarizing stimulus evoking at least 1 action potential. B) Cell by cell analysis of action potential parameters for the action potentials used to compile (A). DMSO caused a negative shift in action potential threshold (P<0.03).

We also analysed if DMSO pre-treatment produced any change in action potential waveform in CA1-PC. This was initially achieved by analysing the waveform of the first action potential evoked by a 300 pA current stimulus (the strongest stimulus applied). Average action potential waveforms are presented in Figure 4A whereas cell by cell analysis of action potential threshold, peak, rate of rise and width at −15 mV are presented for the two groups in Figure 4B. This latter analysis revealed that DMSO pre-treatment altered action potential threshold, moving it nearer to resting potential (P<0.03). None of the other AP parameters were significantly different although the almost 6 mV increase in action potential peak visible in the average waveform of the DMSO pre-treated group just failed to reach significance with a p-value of 0.052(Table 3). In order to confirm these effects on AP properties, the first AP of the first sweep exhibiting at least 1 AP was analysed. This confirmed the observations made when analysing the first AP of the 300 pA sweep (Figure 5).

**Table 3:** A comparison of AP properties in control CA1 pyramidal neurons with those pretreated with DMSO (0.05%).

	DMSO 0.05% n = 24		aCSF n = 20		P
	Mean	SEM	Mean	SEM	
**AP_peak (mV)**	34.5	1.5	28.6	2.6	0.05
**AP_width (ms)**	0.7	0.03	0.7	0.04	0.10
**AP_thres (mV)**	−55.0	0.7	−51.7	1.4	**0.03**
**AP_max_dvdt (Vs^−1^)**	530.4	18.8	482.8	33.7	0.21

### Effects of DMSO on pyramidal cells in perirhinal cortex

Having identified clear effects of DSMO on CA1-PC we were interested to examine if similar or different consequences were seen in a different class of neurone. To this end we again employed brain slice methods to investigate the intrinsic properties of pyramidal neurones in layer 2 of perirhinal cortex (PR-L2PC). Although less widely studied at the cellular level, it is clear that neurophysiologically PR-L2PC behave somewhat differently to CA1-PC [10]. Furthermore, within their overall population, PR-L2PC exhibit greater neurophysiological diversity than CA1-PC. In this study, however, all PR-L2PC cells are presented together, to mirror our analysis of CA1-PC

**Figure 6:**
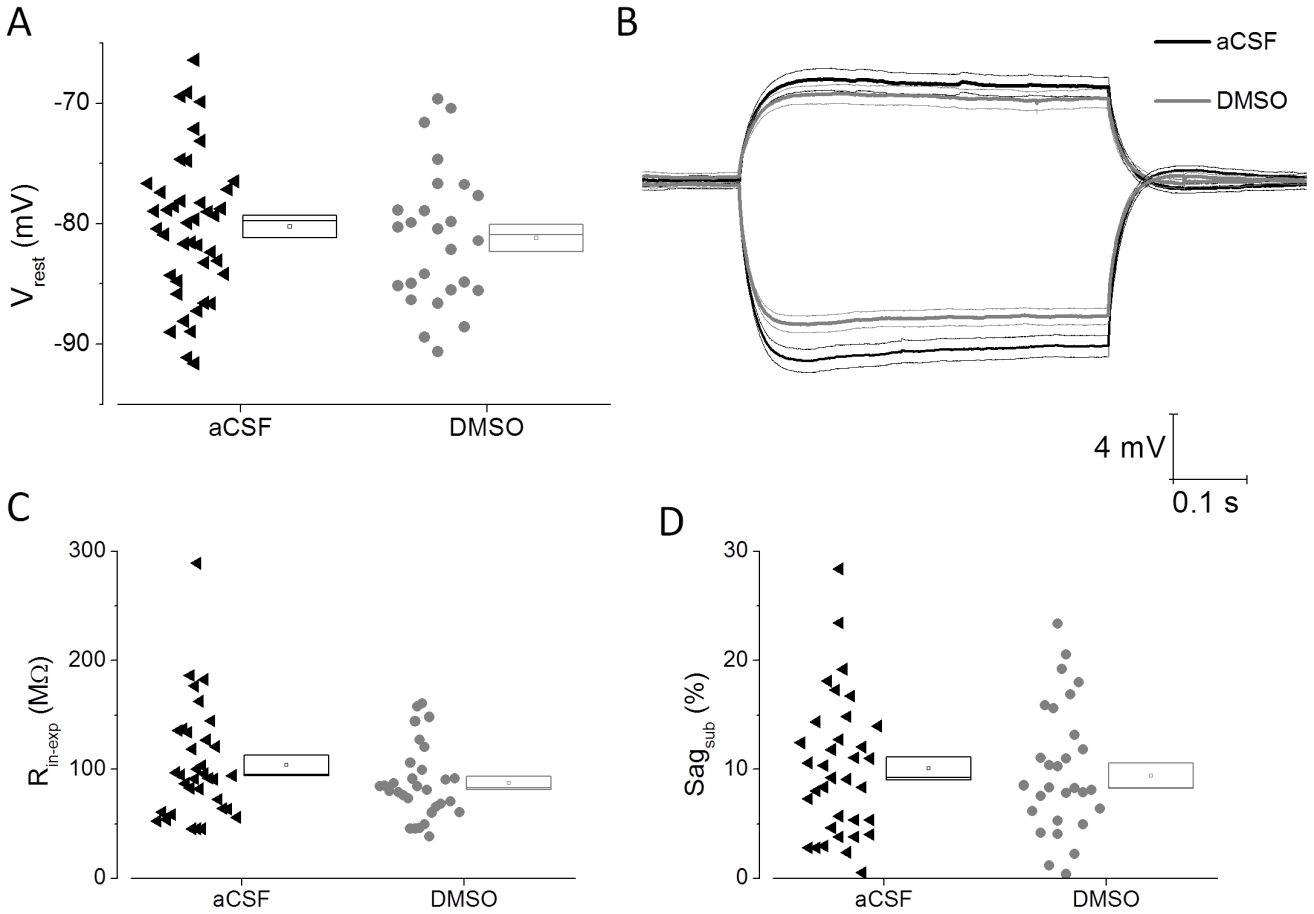
Effects of DMSO pre-treatment on subthreshold intrinsic properties in PR-L2PC. A) A plot of resting potential from 35 control cells (aCSF) and 26 DMSO-treated PR-L2PC. B) Average voltage responses to 500 ms duration −100 and +50 pA current stimuli applied at −80 mV. Data are from 33 control cells (black) and 30 DMSO-treated cells (grey). The thicker central line corresponds to the mean whereas the two adjacent thinner lines represent the bounds encompassed by 1 standard error of the mean. C) Cell by cell analysis of input resistance from PR-L2PC derived from analysis of voltage responses to a −100 pA current stimulus applied at −80 mV. D) Cell by cell analysis of sag in PR-L2PC derived from analysis of voltage responses to a −100 pA current stimulus applied at −80 mV.

Basic sub-threshold intrinsic properties of PR-L2PC are presented in Figure 5. Resting potential was ∼6 mV more negative in control PR-L2PC than CA1-PC, something we have also observed comparing CA1-PC and PR-L2PC in rats (AR, JB and James Cheliah, unpublished observations). As noted in CA1-PC (Figure 1A), there was no difference in resting potential between control and DMSO pre-treated PR-L2PC (Figure 6A), however, paralleling the hippocampal cells (Figures 1 and 2), the average input resistance was lower in DMSO pre-treated PR-L2PC, in this case by around 15%. This effect of DMSO pre-treatment, however, failed to reach statistical significance (P<0.09), possibly due to the greater cell to cell variance within the PR-L2PC population. In control cells, the mean fractional level of sag in PR-L2PC is much less than that in CA1-PC, averaging around only 10%. This is something also seen in rats (AR, JB and James Cheliah, unpublished observations). As expected, the amount of sag was strongly positively correlated with the depolarizing rebound potential seen following cessation of the hyperpolarizing current stimulus (data not shown). Unlike our observations in CA1-PC (Figure 1C) sag did not increase in PR-L2PC after DMSO pre-treatment, indeed the mean level was slightly lower, although not significantly so. Paralleling this, the post-stimulus rebound potential was also slightly reduced after DMSO treatment, although again not significantly so.

**Figure 7:**
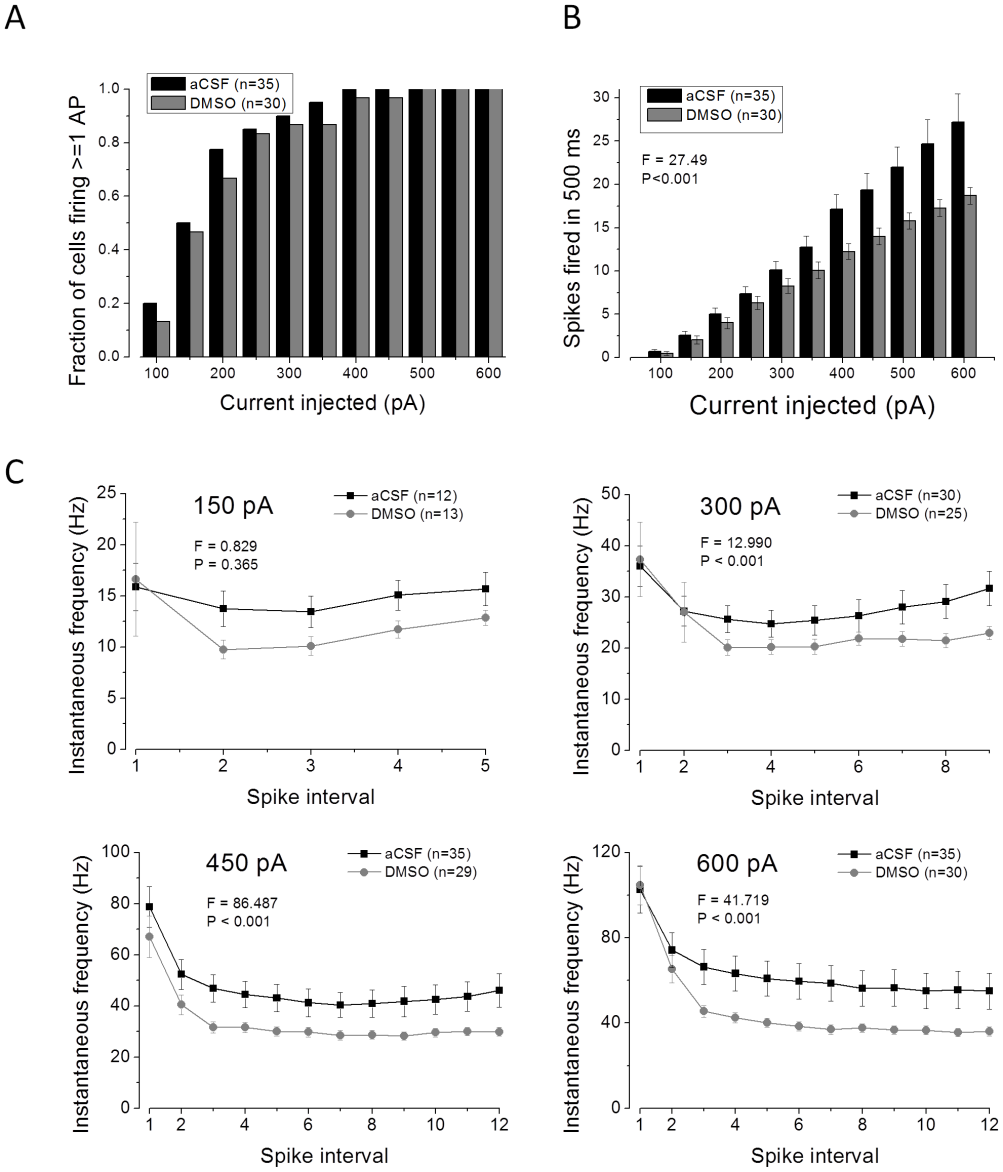
DMSO pre-treatment reduces action potential output in PR-L2PC. A) A plot of the percentage of recordings in which 1 or more action potential was elicited by the various amplitudes of current stimulus indicated on the ordinate. B) A plot of mean number of spikes versus amplitude of current stimulus for control and DMSO pre-treated PR-L2PC. C) Mean instantaneous firing frequencies for 4 different levels of current stimulus ranging from 150 pA (top left) to 600 pA (bottom right).

When PR-L2PC cells at a set membrane potential of −80 mV were challenged with 500 ms depolarizing current pulses (100–600 pA) robust spiking was observed. Similar to CA1-PC, as the stimulus amplitude was increased both the probability of observing firing and the rate of firing increased. A plot of the fraction of cells firing for each level of current stimulus applied is shown in Figure 7A. The mean number of spikes fired for each stimulus is presented in Figure 7B; as in CA1-PC this demonstrates that DMSO pre-treatment reduced action potential output across a range of stimulus intensities (P<0.001, 2 way ANOVA). The temporal dynamics of spiking patterns are presented for four example amplitudes (150, 300, 450 and 600 pA) of 500 ms stimuli in Figure 7C. This indicates that PR-L2PC exhibit near regular spiking with weaker current stimuli but accommodate with stronger stimuli. Furthermore, as in CA1-PC, the post-accommodation steady-state firing rate was consistently reduced when the cells had received DMSO pre-treatment.

**Figure 8:**
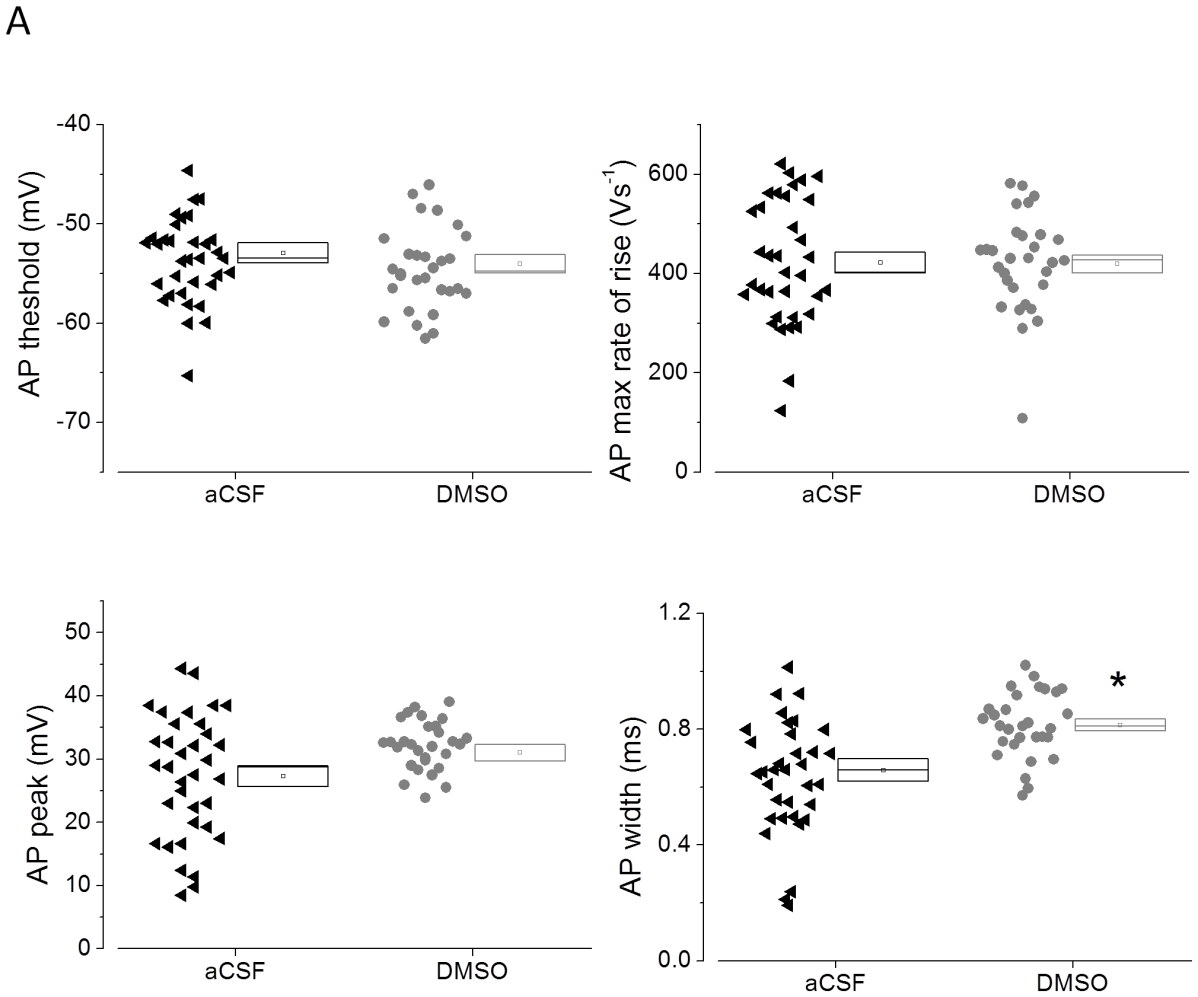
DMSO pre-treatment broadens action potentials in PR-L2PC. A) Cell by cell analysis of action potential parameters from PR-L2PC. Data are compiled from the first action potential produced by a 600 pA depolarizing stimulus applied at −80 mV to 35 control and 30 DMSO-treated cells. DMSO treatment produced a significant increase in action potential width of around 25%.

Waveform analyses of the first action potential produced by a 600 pA current stimulus are illustrated in Figure 8A. As shown for CA1-PC in Figure 4B the graphs present a cell by cell analysis of four different action potential parameters. In contrast to the hippocampal cells a significant broadening of the action potential (P<0.002) was seen in the DMSO pre-treated PR-L2PC, whereas threshold, peak and rate of rise where not altered.

## Discussion

The experiments presented here indicate that *in vitro* incubation of murine brain slices in the aprotic solvent DMSO, at a concentration frequently regarded as biologically innocuous, can produce significant changes to neuronal IE. Effects of DMSO were observed in two different classes of pyramidal neurone, CA1-PC and PR-L2PC. These cells reside in distinct CNS regions, both of which have known roles in cognitive function [3], [4].

In both neurone types the consequences of DMSO exposure can simplistically be regarded as a reduction in excitability, reducing action potential output for any given stimulus (Figures 3C and 6B). This probably for the most part arises from the reduced membrane resistance in DMSO exposed cells, which means any current drive will produce an attenuated voltage response. Notably, however, in CA1-PC, but not PR-L2PC we also observed a negative shift in action potential threshold. This potentially acts to favour action potential generation, and could, under some circumstances, make it easier to elicit action potential generation.

Most importantly these data confirm the need to employ appropriate vehicle controls in experiments using a DMSO vehicle, even at quite modest concentrations. To state the perhaps obvious conclusion, when seeing an effect(s) in response to treatment with an agent applied with DMSO it can only be certain that the effect(s) arise from the agent alone if a comparison is made to the effect of DMSO alone. Beyond this our data tell us that neurones in brain slices are no longer behaving “normally” after a period of exposure to DMSO, although this has to be put within the context of how “normally” neurones behave in an *in vitro* preparation such as a brain slice anyway, something we have discussed previously [11].

It is important to make clear that in these experiments no DMSO was being applied during the electrophysiological recordings themselves. Instead, the solvent treatment was made for 2–5 hours in the holding chamber employed before the slices were transferred to the constantly perfused recording chamber. Furthermore, by the time we established any recording we would expect any residual DMSO to have washed out of the tissue. Consequently the actual recordings were made under identical conditions for both groups. For this reason, the simplest interpretation is the effects of DMSO were already established before the brain slice was transferred to the recording chamber, and also the effects persist at least long enough to outlast the period between placing the slice in the bath and establishing a whole-cell recording, a time which probably varies between 5 minutes and 1 or 2 hours.

By studying the effects of DMSO on two different cell types located in different parts of the limbic system we were able to see if any commonalities arose. Both cell types are classified as pyramidal based on their morphology and both are glutamatergic, however, the intrinsic physiology of CA1-PC and PR-L2PC are somewhat different as can be seen by comparing the component parts of Figures1–5 with those of Figures 6–8. For example, CA1-PC cells have higher resting input resistances, exhibit more HCN-channel mediated sag, and tend to fire more easily and faster typically with a somewhat more “bursty” pattern.

In neither cell type was the resting membrane potential altered by DMSO; this indicates to us that the cells were not in a highly compromised metabolic state, as this would typically produce a substantial depolarization. A similar argument can also be made against gross DMSO-mediated damage to the plasma membrane. DMSO did, however, clearly reduce membrane resistance, in CA1-PC, and a near-significant trend in this direction was also observed in PR-L2PC. Of note in this regard, is the fact that DMSO has been used as a tool to increase cellular permeability [2]. The underlying basis of this decrease in membrane resistance is unknown, although a likely explanation for this observation is that DMSO-treatment increased the number of open ion channels on the membrane. The identity of these channels is not known, although given the lack of change in membrane potential one possible candidate is some form of Cl^-^ conductance, since this would have little effect on resting potential under the ionic conditions employed in these recordings, (i.e. the Cl^-^ equilibrium potential is close to rest). Stimulus-induced increases in surface expression of Cl^-^ channels have certainly been seen in other systems, for example in response to amyloid beta treatment [12]. In parallel to the decrease in R_in_, DMSO appears to cause an inversely proportional increase in membrane capacitance (Cm), this may reflect a change in cell size although our suspicion is this instead reflects a change of the capacitive properties of the bilayer.

A clear change in both cell types studied here was a reduction in the number of spikes fired for a given current stimulus. This could well be a consequence of the altered input resistance, as by Ohm's law the depolarization produced for any given current stimulus would be smaller. More unexpected was the increase in sag conductance seen when −100 pA current stimuli were applied to DMSO pre-treated CA1-PC cells. This was the case for both measures of sag we employed. This outcome is not what would be expected from the reduced input resistance generated in CA1-PC by DMSO, since this would reduce the hyperpolarization produced by the current stimulus and consequently cause activation of fewer HCN channels, the major conductance that produces sag. Instead DMSO pre-treatment appears to be enhancing HCN channel activation. Interestingly, similar concentrations of another low molecular weight polar solvent, namely ethanol, have been reported to enhance gating of both cardiac and CNS HCN channels and thereby to enhance sag [13], [14].

A recent study employing the same mouse strain used here, found that DMSO dosed via the intraperitoneal route to neonatal and young mice caused widespread neuronal apoptosis that developed over a period of a few hours [15]. The anatomical pattern of damage observed changed with age, however, damage to the cortical neuropil was very prominent at all ages. The lowest DMSO dose found to be effective in P7 neonates (0.3 mL/Kg) is roughly similar to the concentration employed here, assuming the agent is equally distributed throughout the body. After 8 hours this dose of DMSO caused a 150% increase in the number of apoptotic cells [15].

In the same study 0.5 and 1% DMSO were shown to kill around 50% of cultured hippocampal neurones prepared from P0-P2 rats, interestingly this effect was absent in cultures in which extracellular potassium was raised to increase neuronal activity [15]. Taken together with our finding that DMSO induces hypoexcitability it is tempting to postulate that changes to IE contribute to DMSO-induced cell damage and death. Notably there is a well-established literature describing the role of K^+^ channels in apoptotic processes [16].

As previously observed, [15] literature on CNS effects of DMSO exposure is very limited, despite the common use of the agent both as a solvent for bioactive molecules and a cryoprotectant. Behavioural effects of DMSO have been reported in adult rats, including actions that might reflect altered cortical and/or hippocampal activity [17], [18]. Importantly significant DMSO exposure is a consequence of stem cell therapies [19] including bone marrow transplantation such as that provided to children with neuroblastoma or Ewing's sarcoma. This raises the possibility of CNS damage as an unwanted consequence of such clinical procedures. Indeed, there are reports that suggest that cryoprotectant DMSO given as a component of stem cell transplants has resulted in neurological damage/outcomes [20]–[24].

Outside of the CNS there have been suggestions that changes to cardiovascular function associated with stem cell infusion may arise from the presence of DMSO [25], [26]. Perhaps the changes to neuronal excitability we describe here have parallels in the excitability of cardiac myocytes which, after all, in the activity of voltage-gated Na^+^, K^+^ and Ca^2+^ channels, have a broadly similar basis to their electrical excitability as neurones.

In laboratory science DMSO is widely used as a solvent for various compounds including pharmacological and biochemical reagents, in particular hydrophobic molecules such as those that can gain access to cell interiors by crossing the plasma membrane. One example of this is the family of fluorescent dyes used to indicate Ca^2+^ concentration inside cells. These molecules, for example Fura-2, are applied to cells in the form of hydrophobic esters that can readily cross cell membranes, following which cellular esterases liberate the active acid moieties that have Ca^2+^-dependent fluorescent properties. These esters are usually dissolved in 100% DMSO and then diluted into aqueous media before applying to tissue of interest for periods of at least 30 minutes and often longer. For loading cellular monolayers the DMSO concentration applied is typically 0.1 to 0.5%, however, even higher concentrations are employed for “the bolus loading methods” used in vivo or in tissue slices [27]. Our data would suggest that these treatments when applied to neurones could be causing long lasting changes to the excitability of the system under investigation.

In summary, pre-treatment with a modest concentration of the solvent DMSO was found to have persistent effects on the excitability of mammalian pyramidal neurones. In future, it will also be important to examine if other classes of neurone, in particular cells which mediate neuronal synaptic inhibition exhibit similar effects. It will also be informative to examine if the neurophysiological changes we describe here are causally linked to the generation of DMSO-induced apoptosis in rodents [15]. Irrespective of such links it is clear that DMSO should be used in experimental science with caution and its various actions considered when introducing this agent into humans for clinical purposes such as stem cell therapy.
